# Decadal demographic shifts and size-dependent disturbance responses of corals in a subtropical warming hotspot

**DOI:** 10.1038/s41598-024-56890-w

**Published:** 2024-03-15

**Authors:** Brigitte Sommer, Jessica M. Hodge, Liam Lachs, James Cant, John M. Pandolfi, Maria Beger

**Affiliations:** 1 School of Life and Environmental Science, The University of Sydney, Sydney, NSW 2006 Australia; 2 School of Biology, Faculty of Biological Sciences, University of Leeds, Leeds, LS2 9JT UK; 3 Maldives Underwater Initiative, Six Senses Laamu, Olhuveli Island, Laamu Atoll Republic of Maldives; 4 School of Natural and Environmental Sciences, Newcastle University, Newcastle upon Tyne, UK; 5 Centre for Biological Diversity, University of St Andrews, St Andrews, KY16 9TH Scotland, UK; 6 School of the Environment, The University of Queensland, Brisbane, QLD 4072 Australia; 7 Centre for Biodiversity and Conservation Science, School of Biological Sciences, University of Queensland, Brisbane, QLD 4072 Australia

**Keywords:** Coral bleaching, Mortality, Population dynamics, Biogeographic transition zone, Bayesian analysis, Chlorophyll *a*, Ecology, Climate-change ecology, Population dynamics, Ecology, Biogeography

## Abstract

Long-term demographic studies at biogeographic transition zones can elucidate how body size mediates disturbance responses. Focusing on subtropical reefs in eastern Australia, we examine trends in the size-structure of corals with contrasting life-histories and zoogeographies surrounding the 2016 coral bleaching event (2010–2019) to determine their resilience and recovery capacity. We document demographic shifts, with disproportionate declines in the number of small corals and long-term persistence of larger corals. The incidence of bleaching (*Pocillopora*, *Turbinaria*) and partial mortality (*Acropora, Pocillopora*) increased with coral size, and bleached corals had greater risk of partial mortality. While endemic *Pocillopora* experienced marked declines, decadal stability of *Turbinaria* despite bleaching, coupled with abundance increase and bleaching resistance in *Acropora* indicate remarkable resilience of these taxa in the subtropics. Declines in the number of small corals and variable associations with environmental drivers indicate bottlenecks to recovery mediated by inhibitory effects of thermal extremes for *Pocillopora* (heat stress) and *Acropora* (heat and cold stress), and stimulatory effects of chlorophyll-*a* for *Turbinaria*. Although our study reveals signs of resilience, it foreshadows the vulnerability of subtropical corals to changing disturbance regimes that include marine heatwaves. Disparity in population dynamics suggest that subtropical reefs are ecologically distinct from tropical coral reefs.

## Introduction

As the global imprint of climate change on natural systems intensifies and environmental conditions are becoming more stressful for many species, demographic approaches are increasingly used to assess the effects of environmental disturbances on the short- and long-term population dynamics of plants and animals^1^. Species respond differently to disturbance, and interspecific and size-dependent variation in demographic performance can provide valuable insights into the mechanisms that underly persistence in different habitats and the idiosyncratic effects of environmental stress^2^. Long-term demographic studies are thus urgently needed to determine taxon-specific responses to disturbance^3^, their recovery capacity^4^ and ultimately to anticipate future community reorganisation under climate change^1^.

Climate change is exacerbating environmental extremes^5^ that often lead to unexpected consequences^6^. Hence, much can be learned from studies of populations that naturally persist in extreme settings^7–9^. Classical demographic theory predicts that natural selection maximises the long-term growth rate of populations in fluctuating environments^10^. Yet, depending on their life history traits, some taxa exploit short-term demographic strategies (e.g., ability to rapidly enhance population growth) to persist in highly variable and extreme environments^11^. Besides, just like species vary in their responses to disturbance, conspecific individuals of different sizes or developmental stages often respond differently to disturbance, thereby influencing population dynamics and demographic resilience^1^. Long-term decadal-scale demographic monitoring is therefore critical to determine recovery trajectories of different species in response to environmental stress, especially for long lived taxa with diverse life histories such as corals^12^ and trees^13^.

Recent evidence suggests that climate-driven disturbances such as drought and marine heatwaves alter size-dependent patterns in mortality, with lasting impacts on recovery capacity^13,14^. Shifts in the size structure of species with contrasting life histories can reflect distinct demographic drivers^4^ and when linked with environmental data can reveal vulnerabilities of distinct life stages to environmental stress^15,16^. Size-based demographic approaches thus provide valuable insights into the potential mechanisms that influence recovery trajectories following disturbance^4,12,17^, such as the existence of recruitment bottlenecks^18^ and post-settlement mortality^19^. For instance, on the Great Barrier Reef (GBR), declines in the number of small corals^20^ and impaired stock-recruitment dynamics have diminished recovery capacity in the years directly following mass coral bleaching in response to heat stress^18^.

Abiotic stress is especially pronounced where species occur at the limits of their geographic distribution and environmental tolerances ^8,21^. As such, subtropical reefs south of the GBR present the ideal system in which to investigate decadal-scale population dynamics of corals in naturally variable and stressful conditions. With above-average observed and projected warming, this region is considered a warming hotspot^22^. Yet, despite tropicalisation of fish assemblages and declines in temperate seaweeds^6^, coral assemblages have remained relatively stable between 1990 and 2013/2014^23^. They subsequently suffered extensive coral bleaching in 2016^16,24,25^ and the extent to which the abundance and size structure of different species have recovered to pre-disturbance trajectories is still unknown but critical for determining their resilience to changing conditions. Moreover, as corals are colonial organisms that can exhibit partial mortality, investigating size-dependent patterns in partial mortality and coral bleaching can provide valuable insights into population dynamics and susceptibilities of different life stages to disturbance^12^.

Here, we quantify decadal patterns in the population size structure of corals in the Solitary Islands Marine Park (30° South) between 2010 and 2019, surrounding the pantropical coral bleaching event of 2016. Specifically, to determine their resilience and recovery potential following heat stress we examine spatio-temporal patterns in the population size-structure of three coral genera with contrasting life-histories and distributions before, during and after coral bleaching. On the Great Barrier Reef, juveniles of most examined coral taxa were more resistant to bleaching than adult corals^26^. Therefore, to test whether corals in high-latitude settings undergo similar size dependency in disturbance responses, we examine whether coral bleaching and partial mortality vary among taxa and are influenced by coral size. Given the importance of population replenishment for recovery and evidence of recruitment limitation in subtropical ecosystems^16,27–29^, we quantify environmental correlates of juvenile coral abundances to determine potential abiotic drivers of recovery dynamics in the subtropics. Consistent with trait-based filtering^30^ and different range limiting factors^21^, we expect that demographic patterns and environmental drivers vary among species and that coral abundance has declined during the 10-year period surrounding the 2016 coral bleaching event. Our study on the long-term population trajectories of different species following heat stress is key to predicting future trajectories of high-latitude reefs in warming seas.

## Methods

### Survey design and data collection

Coral surveys were conducted at four sites in the Solitary Islands Marine Park (30° South) in subtropical eastern Australia between 2010 and 2019. In this region corals persist in cool, light-limited, nutrient-rich and highly variable conditions^21^ and grow directly on rocky substrate. Coral communities exist in pockets of suitable rocky habitat^31^ and are considered marginal and extreme^8^ for most corals. Considering known cross-shelf gradients in biotic communities and environmental conditions in the region^30–32^ we surveyed two inshore (Southwest and Northwest Solitary Islands, 2–6 km from shore) and two offshore (South and North Solitary Islands, 6–11 km from shore) sites located on the semi-protected leeward side of islands (see Appendix Fig. A.1). The four sites were surveyed during the Austral spring of 2010, 2012, 2016, 2018 and 2019, and during a coral bleaching event in April 2016 to monitor temporal change in coral assemblages. As surveys weren’t conducted annually during this period and to investigate the effect of the 2016 bleaching event on coral populations, we grouped the surveys into 3 periods that accounted for the gap in survey years between periods. Specifically, we grouped surveys into Period 1 (2010, 2012), characterising the pre-bleaching state; Period 2 (April and October 2016), encapsulating the bleaching event and immediate aftermath; and Period 3 (2018, 2019), which is long enough after the disturbance to begin to track recovery (i.e., 2–3 years of possible recruitment).

**Figure 1:**
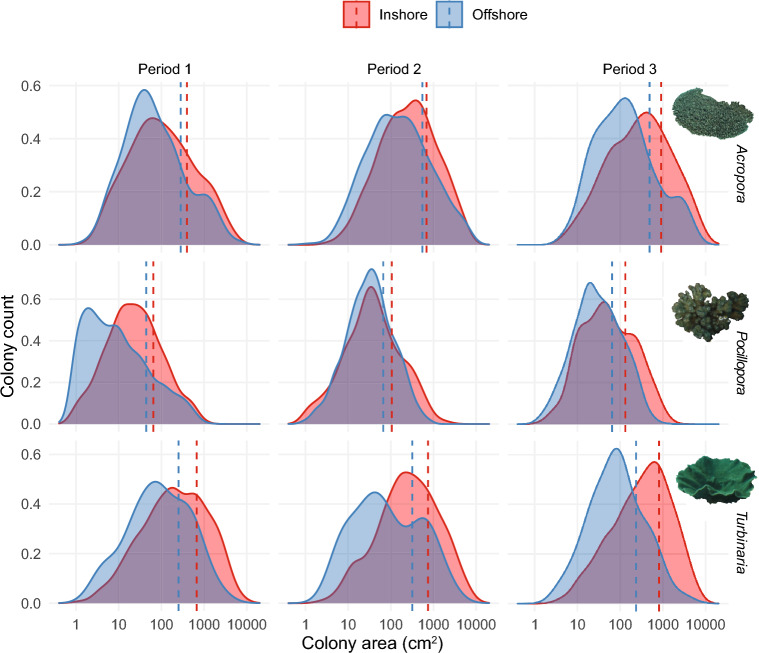
Size-frequency distributions of log-transformed colony area for *Acropora*, *Pocillopora* and *Turbinaria* populations at Inshore (red) and Offshore (blue) sites in the Solitary Islands Marine Park in Period 1 (2010, 2012), Period 2 (April and October 2016) and Period 3 (2018, 2019).

At each site, we ran 3 replicate, 30 m long by 1.2 m wide photographic belt transects at 8-10 m depth, taking 30 downward-facing non-overlapping photos of 1 m × 1.2 m size per transect, capturing a total area of 108 m^2^ across the 3 transects (36 m^2^ per transect) per site. On each visit, transects were haphazardly placed running from an initial marker in the spatially restricted coral habitat (i.e., hundreds of meters), were at least 20 m apart and representative of the study sites^30^. An L-shaped calibration stick was used to maintain consistent vertical distance (80 cm) above the substrate and to include a scale of known size in the photographs for measurements of planar area (see Figure 1 in^33^). Sizes of individual corals in the genera *Acropora*, *Pocillopora* and *Turbinaria* were extracted from photographs using the SizeExtractR workflow in ImageJ and R^33^, recording the genus, and the incidence of bleaching (0 = not bleached, 1 = bleached) and partial mortality (0 = without partial mortality, 1 = with partial mortality) for each coral. Bleaching status and partial mortality were visually assessed from photographs by recording whether a coral had whitened relative to the typical colouration of unbleached *Acropora*, *Pocillopora* and *Turbinaria* corals at the sites, and whether the coral contained dead parts devoid of living tissue, respectively. We chose the three genera due to their high abundance in the region (i.e., up to 70% of Scleractinia abundance^30^) and to determine whether patterns of abundance and population size structure varied among taxa with different distributions and traits: the subtropical endemic *Pocillopora aliciae*, a brooding coral of branching morphology that only occurs in coastal New South Wales (NSW); hermaphroditic, broadcast spawning, predominantly tabular *Acropora* with a tropical-subtropical distribution; and gonochoric, broadcast spawning *Turbinaria* of laminar morphology with a predominantly subtropical distribution.

Although individual corals were not followed through time, we likely captured many of the same coral colonies in our repeat surveys as transects were placed in the same area on each visit to limit within-site variability. To ensure consistent capture of small corals across surveys, we applied a lower size cut-off of 0.8 cm^2^ (~ 1 cm diameter), excluding all corals < 0.8 cm^2^ from data analysis. 10.7% of coral colonies were not fully captured in the photographs and we were only able to outline the perimeter of the colony area visible in the photographs for these corals as information from outside the photographs was unavailable. To ensure that the underestimated sizes of these partially captured colonies did not affect our results we performed analyses with and without partially captured colonies. As we found no qualitative difference in the results and because the inclusion of partially captured colonies provides more accurate estimates of coral abundance, all analyses in the main manuscript include partially captured colonies. Complementary analyses that exclude partially out of frame corals can be found in Appendix A and show that sub-setting of the data did not alter the main findings.

### Environmental data

The physical and chemical oceanography of this region is strongly influenced by the warm poleward flowing East Australian Current (EAC), with greater EAC influence at offshore islands where temperatures are approximately 1 °C higher^32^ and chlorophyll *a* concentrations are lower than at inshore sites^34^. EAC waters are generally more oligotrophic and have lower chlorophyll *a* concentrations than adjacent Tasman Sea Water^35^, also affecting light penetration and water clarity. To characterise environmental conditions at the study sites and examine their influence on coral population replenishment, we compiled daily sea surface temperature data (SST) from the global 5 km resolution NOAA Coral Reef Watch CoralTemp reanalysis^36^ and daily chlorophyll *a* (Chla) data at 9 km resolution from NASA OceanColour^37^ as a proxy for nutrient content. Temporal gaps in the Chla record were filled using linear interpolation. We calculated mean annual SST (SST_mean) and Chla (Chla_mean) for each site and year, as well as three thermal stress metrics to examine the role of heat and cold stress. Specifically, we calculated Degree Heating Weeks (DHW_0C_) as the accumulated weekly temperature anomalies (daily data divided by 7 to move from days to weeks) over the previous 12 weeks (i.e., 84 days inclusive) that exceed the long-term maximum of monthly means climatology (MMM) to account for the accumulation of low-level heat stress following^16,24,38^. This means that the absolute values of DHW_0C_ are greater than the conventional NOAA Coral Reef Watch metric that accumulates thermal anomies greater than 1 °C above the MMM^24,36,38^. Analogous to this definition, we calculated Degree Cooling Weeks (DCW) as the accumulation of values below the long-term minimum of monthly means (mMM) over the preceding 12-week period^39^, whereby larger negative values indicate higher accumulated cold stress (DCW_0C_). As cold stress is an important predictor of coral biodiversity patterns in this^21^ and other high-latitude regions^40,41^, we computed a second DCW metric with a higher temperature threshold to assess the potential effect of very low magnitude cold stress at the poleward range-limit of corals (DCW_1C_). In the computation of DCW_1C_ cold stress starts accumulating once SST reaches a threshold of 1 °C above the mMM (i.e., once SST is colder than mMM + 1 °C).

Data analyses.

### Taxonomic, spatial and temporal trends in colony size structure

To examine spatiotemporal trends in the colony size structure of corals with contrasting life histories we constructed size-frequency distributions for *Acropora*, *Pocillopora* and *Turbinaria* corals based on log-transformed colony area. We used 2-sample Kolmogorov–Smirnov tests to examine whether size-frequency distributions varied among taxa, periods and between inshore and offshore sites.

To further characterise size-based patterns, we divided colony counts into five size classes (quintiles), which we grouped into small (1st quintile), medium-sized (2nd to 4th quintile) and large (5th quintile) corals following Dietzel, et al.^20^. The abundance of small corals is a proxy for population replenishment, encompassing variation in both settlement and post-settlement survival and the abundance of large corals can characterise reproductive output. As population size structure tends to vary among taxa and habitats^42^, the boundaries between size bins were allowed to vary among taxa and habitats (i.e., inshore, offshore), but fixed across years. We also calculated mean colony size, coefficient of variation, skewness, and the 1st and 5th quintiles of colony size. The coefficient of variation (CV = σ⁄μ) is a standardised measure of the dispersion of size-frequency distributions suited to comparing populations with different mean colony sizes. Skewness measures asymmetry of size-frequency distributions and reflects the proportion of small versus large colonies, whereby positive and negative skewness indicate a high proportion of small and large corals, respectively^42^.

To characterise spatial and temporal trends in the colony size structure of *Acropora*, *Pocillopora* and *Turbinaria* corals in detail, we calculated changes in the abundance of small (1st quintile), medium (2nd to 4th quintile) and large (5th quintile) corals at inshore and offshore sites between periods 1 (2010, 2012) and 3 (2018, 2019) following Dietzel, et al.^20^. We also determined changes in the mean and CV of colony size, and of the 1st and 5th quintile of the colony size structure to assess changes in the size of the smallest (bottom 20%) and largest (top 20%) corals in the population, respectively. Specifically, an increase in the 1st quintile indicates that corals in the bottom 20% were larger due to a decline in the relative abundance of small corals. An increase in the 5th quintile indicates a size increase of corals in the 80th percentile due to an increase in the relative abundance of large corals. We used bootstrap resampling (n = 1000) from posterior distributions to characterise uncertainties in size-class abundances and report parameter uncertainties as 66% and 95% highest posterior density intervals.

We used Bayesian generalised linear models (GLMs) to assess the interactive effects of period, shelf position and taxa (~ period * position * taxa) on trends in mean coral size (family = gaussian) and coral abundance (family = log link negative binomial) pooled across transects. We then used Bayesian binary logistic regression (family = bernoulli) to assess the effects of coral size (logArea) and taxon on whether corals bleached (bleaching ~ logArea * taxa) or experienced partial mortality (partial mortality ~ logArea * taxa) and to determine whether the odds of corals suffering partial mortality varied among periods and taxa (partial mortality ~ period * taxa). We also tested whether bleached corals had greater odds of suffering partial mortality than unbleached corals (partial mortality ~ bleaching; family = bernoulli).

### Environmental correlates of patterns in the abundance of small corals

The recruitment of new individuals is key to population recovery after disturbance, and in open marine populations, captures both pre- and post-settlement processes^43^. In coral surveys, the abundance of small corals is frequently used as a proxy for recruitment^19,20,44^ encapsulating processes not directly measured in the surveys that can affect recovery, such as larval supply, settlement rates and post-settlement survivorship^43,45^. To investigate how abiotic conditions might be influencing population replenishment on subtropical reefs, we therefore fit Bayesian GLMs with a log link negative binomial variance structure to examine the environmental predictors (see Table A.3. for predictors in the models) that best explained spatiotemporal trends in the abundance of small *Acropora*, *Turbinaria* and *Pocillopora* corals as a proxy for recruitment. Notably, small colonies can also come about thorough partial mortality, however, the incidence of partial mortality was low in our study. Due to multi-collinearity (r > 0.8, Appendix Table A.2), the DCW_1C_ metric was used in the models to assess the effect of cold stress. We calculated Bayes R^2^ values and used approximate leave-one-out cross-validation with LOO information criterion (LOOIC) to assess model fit and compare model weights (i.e., the relative likelihood of a model, based on the relative scaling of models in the model set).

All models were created using the probabilistic framework offered by Bayesian statistics and executed in Stan, accessed with the R package ‘brms’^46^. We chose a Bayesian approach as it (i) quantifies uncertainty by sampling from the posterior distributions, (ii) can account for asymmetrically distributed uncertainty distributions, and (iii) flexibly handles complex models that quantify uncertainty for all parameters^46^. We summarised model fits using the 95% highest posterior density interval as the credible interval and computed median point estimates for all chains. To improve convergence and guard against overfitting, we specified weakly informative conservative priors and ran each model with three chains of 4000 iterations (warmup = 200) and a thinning rate of 5. We examined chain mixing, performed posterior predictive checks to assess model fit, and used the Gelman–Rubin convergence diagnostic (R-hat) to assess model convergence.

## Results

### Patterns in coral size structure

**Figure 2:**
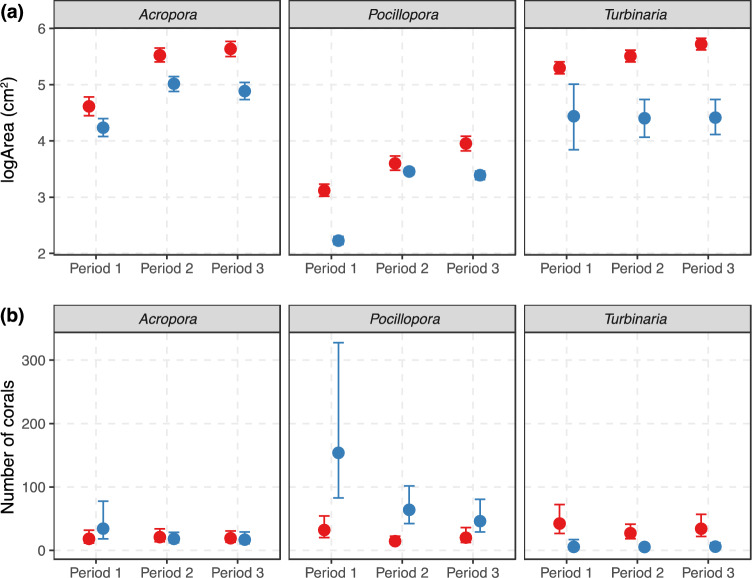
Patterns in the (**a**) mean size and (**b**) number of *Acropora*, *Pocillopora* and *Turbinaria* corals at Inshore (red) and Offshore (blue) sites in the Solitary Islands Marine Park in Period 1 (2010, 2012), Period 2 (April and October 2016) and Period 3 (2018, 2019). Estimates are shown as 95% highest posterior density intervals with 95% upper and 95% lower credible intervals.

We recorded a total of 13,195 coral colonies across 2160 images; 6992 *Pocillopora*, 3207 *Turbinaria* and 2996 *Acropora*, with *Pocillopora* more abundant offshore and *Turbinaria* more abundant inshore (Figs. 1 and 2, Table A.1). Size-frequency distributions varied markedly among taxa (Fig. 1), with *Pocillopora* and *Turbinaria* (2-sample KS test, D = 0.517, *p* < 0.001) most dissimilar, followed by *Pocillopora* and *Acropora* (D = 0.243, *p* < 0.001) and *Acropora* and *Turbinaria* (D = 0.112, *p* < 0.001) for all sites and years pooled. Log-transformed size-frequency distributions were negatively skewed for *Turbinaria* (− 0.29), indicating relatively fewer colonies in the smaller size classes and a dominance of large corals. Colony size structure of *Pocillopora* (− 0.03) and *Acropora* corals (0.05) was fairly symmetrical (i.e., skewness close to zero) when pooled for all sites and years (Appendix Table A.1).

Size-frequency distributions varied significantly between inshore and offshore habitats for all genera (2-sample KS test, *Turbinaria*, D = 0.308, *p* < 0.001; *Acropora* D = 0.161, *p* < 0.001; *Pocillopora*, D = 0.125, *p* < 0.001), with higher densities of larger corals inshore (Fig. 1). Colony size varied most for *Pocillopora* (CV = 0.51) and least for *Turbinaria* corals (CV = 0.31, Appendix Table A.1). Size-frequency distributions changed over time for all taxa. Notably, over the duration of the study (i.e., Periods 1 vs. 3) temporal shifts were most pronounced for *Pocillopora* (Offshore D = 0.367, *p* < 0.001; Inshore D = 0.216, *p* < 0.001) and *Acropora* (Inshore D = 0.249, *p* < 0.001; Offshore D = 0.189, *p* < 001), with *Turbinaria* populations the most stable (Inshore D = 0.129, *p* < 0.001; Offshore D = 0.129, *p* = 0.74). Greatest shifts occurred between Periods 1 and 2 (*Pocillopora*, Offshore D = 0.4, *p* < 0.001, Inshore D = 0.15, *p* < 0.001; *Acropora*, Inshore D = 0.238, *p* < 0.001, Offshore D = 0.219, *p* < 0.001; *Turbinaria* Inshore D = 0.066, *p* = 0.023, Offshore D = 0.124, *p* = 0.765), with minor changes in size-frequency distributions between Periods 2 and 3 for *Pocillopora* (Inshore D = 0.099, *p* = 0.016; Offshore D = 0.059, *p* = 0.014) and *Turbinaria* Inshore (D = 0.084, *p* = 0.002).

**Figure 3:**
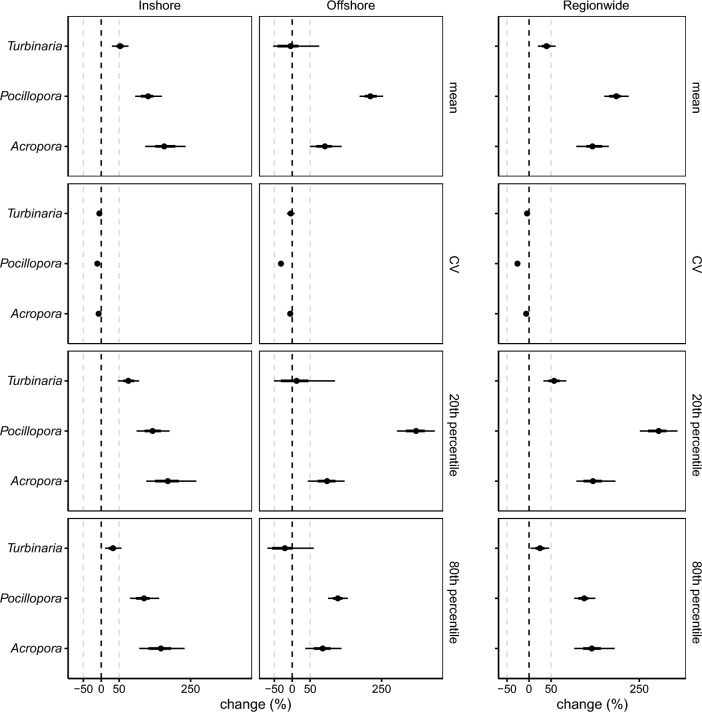
Changes in the mean, coefficient of variation (CV) and 20th and 80th percentile of the colony size structure of *Acropora*, *Pocillopora* and *Turbinaria* corals in the Solitary Islands Marine Park between Period 1 (2010, 2012) and Period 3 (2018, 2019). Percentiles are indicators for the relative abundance of the smallest (20th percentile) and largest (80th percentile) corals, where increases in the 20th and 80th percentiles indicate a decrease in the relative abundance of the smallest corals and an increase in the relative abundance of largest corals in the population, respectively. All estimates are shown as 95% highest posterior density intervals where the point indicates the median, the thick line the 66% credible interval and the thin line the 95% credible interval.

Mean colony size varied among taxa and ranged between 66.42 cm^2^ for *Pocillopora* and 708.47 cm^2^ for *Turbinaria* and was larger inshore for all taxa during all time periods (Fig. 2, Appendix Table A.1). Colony size structure shifted towards larger colonies through time, as shown by increases in mean colony size for all taxa as well as increases in the size of small (in the 20th percentile) and large (in the 80th percentile) colonies and declines in the coefficient of variation (Fig. 3).

### Patterns in coral abundance

**Figure 4:**
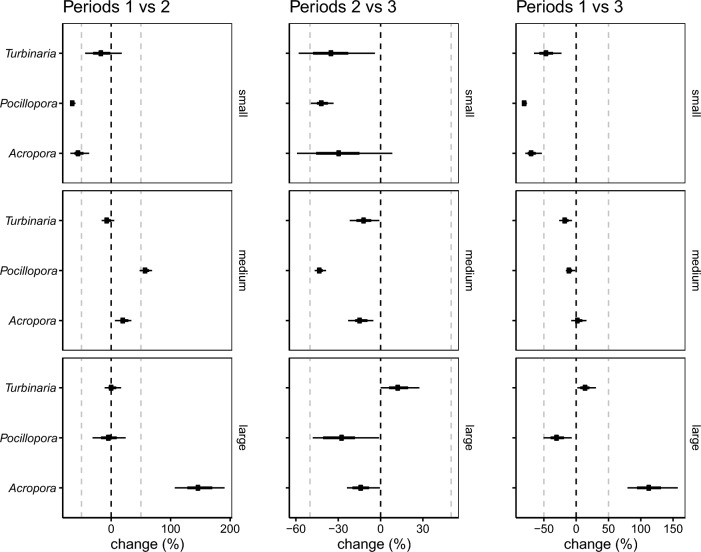
Changes in the abundance of small, medium, and large colonies for all taxa between Periods 1 versus 2, Periods 2 versus 3, and Periods 1 versus 3. Percentage changes in abundances are defined as changes in the number of corals in the 1st quintile (small), 2nd–4th quintile (medium) and 5th quintile (large) of colony size. All estimates are shown as 95% highest posterior density intervals. The point indicates the median, the thick line the 66% credible interval and the thin line the 95% credible interval.

The abundance of *Pocillopora* colonies declined through time, especially offshore, whereas the abundance of *Turbinaria* and *Acropora* remained relatively stable (Fig. 2b). The abundance of small corals declined for all taxa in all periods (Fig. 4). Although the abundance of medium and large corals followed a stable to upward trajectory up until 2016 (Periods 1 vs. 2), the abundance of medium and large corals declined considerably in the aftermath of coral bleaching (Period 2 vs. 3) for all taxa, except for slight increases in the abundance of large *Turbinaria* colonies (Fig. 4).

### Patterns in coral bleaching and partial mortality

**Figure 5:**
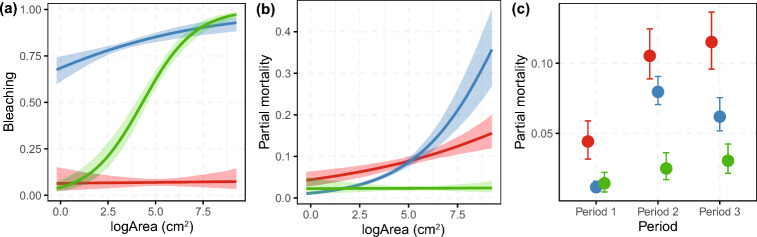
Relationships between the size of *Acropora* (red), *Pocillopora* (blue), and *Turbinaria* (green) corals and the probability of suffering (**a**) coral bleaching and (**b**) partial mortality. (**c**) Temporal patterns in the probability of partial mortality for Period 1 (2010, 2012), Period 2 (April and October 2016) and Period 3 (2018, 2019). Estimates are shown as 95% highest posterior density intervals with 95% upper and 95% lower credible intervals.

The incidence of coral bleaching varied among taxa, with the probability of bleaching highest for *Pocillopora* (0.806), followed by *Turbinaria* (0.653) and minimal bleaching of *Acropora* (0.069). Larger corals had a higher probability of bleaching (Fig. 5a), and this effect was greatest for *Turbinaria*, followed by *Pocillopora* and was not significant for *Acropora* (95% CI − 0.17, 0.20 overlapped zero).

Coral size also influenced whether a coral suffered partial mortality, with larger *Acropora* and *Pocillopora* corals exposed to greater odds of partial mortality (Fig. 5b). *Acropora* corals had the highest probability of suffering partial mortality across all periods (0.04 Period 1, 0.106 Period 2, 0.116 Period 3; Odds of 2.581 and 2.851 compared to 0.04 in Period 2), followed by *Pocillopora* (0.012 Period 1, 0.079 Period 2, 0.062 Period 3) and *Turbinaria* (0.014 Period 1, 0.0251 Period 2, 0.03 Period 3). The odds of suffering partial mortality were higher in Periods 2 and 3, compared to Period 1 for all taxa (but not significant for *Turbinaria*) (Fig. 5c). Bleached corals had a greater probability of partial mortality (0.0552) than unbleached (0.0183) corals (95% CI 1.62, 4.66).

### Environmental correlates of patterns in the abundance of small corals

**Figure 6:**
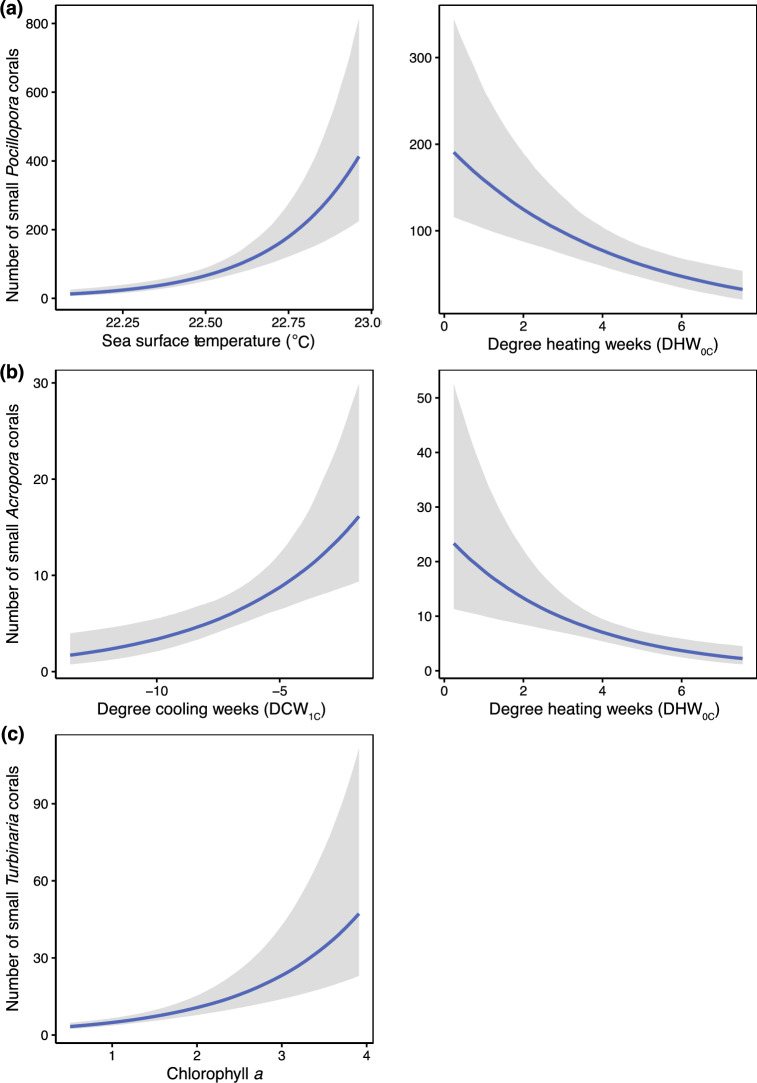
Relationships between the number of small corals and environmental conditions on subtropical reefs in the Solitary Islands Marine Park, eastern Australia, for (**a**) *Pocillopora*, (**b**) *Acropora,* and (**c**) *Turbinaria* corals. Plots show best models for results given in Supplementary Table A.3. Note that for Degree Cooling Weeks, larger negative values indicate higher accumulated cold stress. Estimates are shown as 95% highest posterior density intervals with 95% upper and 95% lower credible intervals.

We found spatio-temporal variation in environmental conditions, with higher SST_mean and DHW_0C_ offshore, and higher Chla_mean and DCW_1C_ inshore (Figure A.3). Low-level heat and cold stress accumulated in all survey years, with highest heat stress (DHW_0C_ = 7.54 °C weeks at North Solitary Island) in 2016 (i.e., the bleaching year) and highest cold stress (DCW_1C_ = 13.52 °C weeks at Southwest Solitary Island) in 2012 (Figure A.3). Environmental predictors of the abundance of small corals varied among taxa (Fig. 6, Table A.3). Specifically, the abundance of small *Acropora* corals was best explained by patterns in thermal stress, with small corals more prevalent in areas (or times) of low heat (DHW_0C_) and cold stress (DCW_1C_). Small *Pocillopora* corals were more abundant where SST_mean was high and DHW_0C_ was low. The abundance of small *Turbinaria* corals was independent of thermal stress and showed a positive association with Chla_mean (best model). The second-best model for *Turbinaria* indicated more small corals where SST_mean was low and Chla_mean was high (model weight = 0.2; Table A.3).

## Discussion

Climate change poses a significant threat to biodiversity across natural systems globally^47^. In coral reef ecosystems, recurrent mass bleaching episodes have led to dramatic loss of coral in response to heat stress^25^. Here, contrary to wholesale declines in coral populations across taxa, size classes and habitats recorded on the GBR^20^, we found more nuanced and optimistic results in a subtropical warming hotspot south of the GBR. Although endemic *Pocillopora* experienced marked declines in the decade surrounding the 2016 bleaching event, decadal stability of *Turbinaria* despite high incidence of bleaching, coupled with abundance increase and bleaching resistance observed in *Acropora* indicate remarkable resilience of these taxa in the subtropics. Shifts away from small corals point to bottlenecks to population replenishment for all taxa^16,48^ with abundance patterns of small corals best explained by thermal extremes (*Pocillopora*, *Acropora*) and patterns in chlorophyll *a* concentrations (*Turbinaria*). Nevertheless, persistence of larger corals despite higher incidence of bleaching and partial mortality, could boost recovery due to disproportionate contribution of large corals to population growth rates via survival, colony growth^49^ and reproduction^18^. Our findings support the notion that subtropical reefs are ecologically distinct from tropical coral reefs^30,50,51^, highlighting disparity in population dynamics^11^ and bleaching responses^24,48^ compared to tropical coral reefs.

### Patterns in coral abundance

While we recorded declines in the abundance of small corals for all taxa and periods (declines of *Turbinaria* in Period 1 vs. 2 and *Acropora* in Period 2 vs. 3 based on 66% credible intervals), the number of medium and large corals followed a stable to upward trajectory for all taxa up until 2016, consistent with corals growing into larger size classes. In the aftermath of coral bleaching, the abundance of medium and large corals declined, apart from slight abundance increases of large *Turbinaria* corals (Fig. 4). This pattern likely reflects disproportionate bleaching and partial mortality of larger corals (Fig. 5). The decadal declines in the number of small corals reported here mirror long-term declines in the abundance of small corals on the GBR^20^ and are consistent with recruitment limitation and post-settlement mortality on high-latitude reefs^28^. Indeed, multiple lines of evidence point to recruitment bottlenecks on subtropical reefs in eastern Australia, ranging from settlement studies using tiles^29^ to size-based demographic studies^16,27,48^ and dispersal models that show limited connectivity with the southern GBR at spawning time^23^. Moreover, disturbance can cause high post-settlement mortality of early recruits^14,15^, especially when impacted by multiple disturbances such as coral bleaching and storms^52^, and in systems with high abundance of macroalgae^53^. Subtropical reefs are naturally exposed to marginal conditions^8,21,50^ that are further exacerbated by extreme storm events, and recently also by heat stress^16,24^. It is thus plausible that these factors, in combination with high abundance of macroalgae and sessile invertebrates^31,50^ limit coral establishment and impair recovery following disturbance.

### Variable associations with environmental drivers

Variable links between the abundance of small corals and environmental drivers across taxa shows that in the subtropics, corals are exposed to complex multi-stressor regimes, particularly extremes in temperature and eutrophication. Indeed, negative association of the number of small *Acropora* corals with both degree cooling and heating weeks, illustrates their sensitivity to both cold and heat stress at high latitudes, where DCW_1C_ reached 13.52 °C in 2012 and DHW_0C_ reached 7.54 °C weeks in 2016. The number of small *Pocillopora* corals was positively associated with higher average temperatures, but still sensitive to accumulated heat stress (DHW_0C_). This vulnerability to heat stress is consistent with high incidence of bleaching of the subtropical endemic *Pocillopora aliciae* recorded here and by Lachs et al.^16^, and of *Pocillopora* species in the subtropical region more broadly^24^. Notably, the abundance of small *Turbinaria* corals was best explained by gradients in chlorophyll *a*, with small corals faring better in high-nutrient conditions (Fig. 6). These taxon-specific results highlight nuanced relationships with varying environmental conditions in this region, likely mediated by inhibitory effects of thermal extremes for *Pocillopora* and *Acropora*, and by stimulatory effects of food availability in higher chlorophyll *a* conditions for *Turbinaria* via heterotrophic feeding^54,55^.

While the role of thermal stress on coral biology and ecology is well established, other factors such as food availability are less often explored. Yet, heterotrophic feeding and trophic plasticity are important when it comes to regulating persistence in abiotically stressful conditions^55,56^. For example, heterotrophic feeding increases resilience to coral bleaching in turbid^7^ and chlorophyll *a* environments (e.g., in Yap, Micronesia, where eddies enhance productivity on the leeward side of islands)^57^, boosts reproductive output^58^, and influences calcification and growth rates^55^. The capacity to exploit these strategies relies on food availability, which tends to be higher in nutrient-rich settings such as turbid and high-latitude environments. Indeed, Fox et al.^54^ found that many corals increase heterotrophy as a function of food availability. In Bremer Bay, Western Australia (34.3° S) for instance, calcification rates for *Turbinaria reniformis* were elevated when chlorophyll *a* concentrations were higher during winter, despite cooler temperatures^59^. On mesophotic high-latitude reefs at Rottnest Island (32° S), *Coscinaraea marshae* corals that survived in a bleached state for more than 11 months showed signs of increased heterotrophic feeding^60^. The relative stability of *Turbinaria* abundance over our 10-year study, despite high incidence of coral bleaching, also supports the positive role of food availability on persistence of *Turbinaria* corals in subtropical environments. Nevertheless, phytoplankton blooms (typically measured as chlorophyll *a* concentrations) can also impose nutrient stress on corals and negatively affect their physiological performance, such as leading to reduced reproductive success, calcification, or linear extension^61^. These effects vary among species and might contribute to variation in demographic structure among taxa and between inshore and offshore sites (Figs. 3, 4, and 5), probably linked to variable encroachment of the EAC and subtle differences in environmental regimes^32,34,35^.

### Taxonomic and size-based responses to disturbance

Subtropical reefs are sometimes heralded as thermal refugia for tropical species in warming seas, yet mounting evidence shows that they are also vulnerable to coral bleaching^24,62–65^ and future climate risks^66^. In this study, the subtropical endemic *Pocillopora alicae* had the highest incidence of coral bleaching, followed by *Turbinaria* and minimal bleaching of *Acropora*. This is consistent with other studies, showing that genera normally among the first to bleach in the tropics (e.g., *Acropora*) were less vulnerable to bleaching in marginal or extreme environments^7^. In the subtropics, endemic species, subtropical specialists^24^ and species close to their northern distributional limits^65^ typically have higher rates of bleaching, possibly because they live close to their upper thermal limits.

Body size influences the physiology, demography and ecology of organisms including how they respond to disturbance^13,67^. For example, mortality from extreme drought disproportionately affects larger trees^13^ and corals of certain growth forms (e.g., tabular corals) become increasingly susceptible to hydrodynamic dislodgement as they grow^67^. We have found size-dependent incidence of coral bleaching and partial mortality in the subtropics (Fig. 5). Specifically, larger *Turbinaria* and *Pocillopora* colonies had a higher probability of bleaching than smaller colonies. This is consistent with efficient mass transfer in small corals^68^ and positive size-bleaching relationships in experimental and field studies from subtropical Japan^68^, the Mediterranean^69^, French Polynesia^14^, and the Great Barrier Reef^26^, but in contrast with *Pocillopora meandrina* populations in Hawaii that showed the opposite pattern^70^. Differences in the shape of these relationships (Fig. 5) are likely due to disparity in the bleaching severity between *Pocillopora* and *Turbinaria* species, with severe and mild bleaching of these taxa, respectively^24^.

Furthermore, we found that larger *Acropora* and *Pocillopora* corals had greater odds of partial mortality than smaller ones, consistent with patterns found on disturbed reefs in the Caribbean^42^ and the general expectation that rates of partial mortality increase with colony size^12^. Unsurprisingly, bleached corals had a greater probability of partial mortality compared to unbleached corals, and the odds of suffering partial mortality were higher in the latter periods compared to Period 1 for all taxa (but not significant for *Turbinaria*). This points to elevated physiological stress following coral bleaching and is consistent with higher incidence of partial mortality after coral bleaching in the Galapagos Islands^71^.

### Implications

The post-bleaching effects on coral populations may not become evident until years after the event^72^. For instance, mortality of adult size-classes can lead to collapse in recruitment in the years following bleaching^18^, and recovery can be suppressed for several years under chronic stress^73^. Moreover, sublethal stress can depress growth rates^74^ and fecundity^18,75^ of surviving corals, with negative effects on population growth and increases in mean colony size^12,20^. Indeed, this is likely to have occurred at the Solitary Islands Marine Park, as supported by declines in the number of small corals and increases in mean colony size for all taxa post bleaching. This suggests that coral recovery in the Solitary Islands was incomplete during the three-year period of data collection following bleaching, consistent with GBR recovery projections of at least seven years (to 70% of pre-disturbance levels) in the absence of disturbance^73^.

While some studies have reported the selective loss of larger corals and declines in mean colony size after disturbance (reviewed in^12^), we found the opposite pattern in the subtropics, consistent with long-term stability of large corals on high-latitude reefs in South Africa^28^. Indeed, the decadal stability of *Turbinaria* populations despite high incidence of coral bleaching (0.653 probability), and the abundance increase and bleaching resistance of *Acropora* corals recorded in this study point to remarkable resilience of these taxa in the subtropics. This supports the results of coral cover-based studies (i.e., not accounting for the size of individual corals) that predated the 2016 bleaching event and showed long-term stability of coral assemblages despite warming in this region^23^. Notwithstanding the loss of *Pocillopora* corals recorded here and the absence of heat stress, we predict that remnant *Pocillopora* will undergo recovery in the coming years, especially in light of their brooding reproduction and higher reproductive success in the region compared to other taxa^29^, properties that enhance their ability to increase population growth following disturbance (i.e., their capacity for demographic compensation)^11^.

Although our study reveals some signs of coral resilience in this warming hotspot, it also underlines the vulnerability of subtropical coral populations to changing environmental conditions. Population decline is inevitable without recruitment and, alarmingly, all three taxa suffered decadal declines in the number of small corals, likely due to recruitment and mortality bottlenecks of early life-stage corals. Recruitment limitation could thus hamper population replenishment following disturbance. While subtropical coral populations appear to be well adapted to the dynamic multi-stressor regime at high latitudes, taxon and size-specific bleaching and mortality indicate nuanced responses to heat stress and foreshadow vulnerability to changing disturbance regimes that include more frequent or severe heat stress events. Indeed, although population declines were greatest for *Pocillopora* corals in this study, projected long-term stochastic population growth trajectories suggest that the abundance of *Pocillopora*, *Acropora* and *Turbinaria* corals could drastically decline by 2100 under escalated future thermal stress in this region^48^. Nevertheless, the observed stability of large corals (Figs. 3 and 4) during the study period, despite higher incidence of bleaching and partial mortality, could bolster recovery because the largest corals in a population contribute disproportionately to reproduction^76^ and population growth^49^. Moreover, in these rocky reef environments where many coral species have massive and flat growth^30^, large branching *Pocillopora*, laminar *Turbinaria* and tabular *Acropora* corals provide structurally complex habitats for fish and invertebrates and could thus enhance associated biodiversity^77^. Larger corals are less likely to completely fit into our photographs than smaller corals and possibly bias our population size structure estimates towards smaller corals (see discussion in^78^). The observed decadal shift towards larger colonies and the positive scaling of disturbance responses with coral size are therefore likely conservative. Our study highlights the utility of long-term demographic studies to determine taxon and size-specific responses to changing disturbance regimes in marginal environments and implications for population recovery. It also emphasises the need for further studies into larval dispersal^23^ and the factors that influence settlement dynamics and success of early life-stage corals^4,15,19,41,79^ in this region to predict future population trajectories.

### Supplementary Information

Supplementary Information.

## Data Availability

Data used in this study and additional information are available upon request from the authors.
