# Supplementary material for: Decadal demographic shifts and size-dependent disturbance responses of corals in a subtropical warming hotspot
Source: Sci Rep. 2024 Mar 15;14:6327. doi: 10.1038/s41598-024-56890-w (PMC10943097; doi:10.1038/s41598-024-56890-w)
Supplement: Supplementary file 1 — Supplementary Information. [file 41598_2024_56890_MOESM1_ESM.pdf]

Appendix A - Decadal demographic shifts and size-dependent disturbance responses of corals in a subtropical warming hotspot

Brigitte Sommer [1*], Jessica M. Hodge [2,3], Liam Lachs [4], James Cant [2,5], John M. Pandolfi [6], Maria Beger [2,7]

[1] School of Life and Environmental Science, The University of Sydney, Camperdown, NSW 2006, Australia.

[2] School of Biology, Faculty of Biological Sciences, University of Leeds, Leeds, LS2 9JT United Kingdom.

[3] Maldives Underwater Initiative, Six Senses Laamu, Olhuveli Island, Laamu Atoll, Republic of Maldives

[4] School of Natural & Environmental Sciences, Newcastle University, Newcastle upon Tyne, UK

[5] Centre for Biological Diversity, University of St Andrews, KY16 9TH, Scotland, UK

[6] School of the Environment, The University of Queensland, Brisbane, QLD 4072, Australia.

[7] Centre for Biodiversity and Conservation Science, School of Biological Sciences, University of Queensland, Brisbane, QLD, 4072 Australia

* Corresponding author

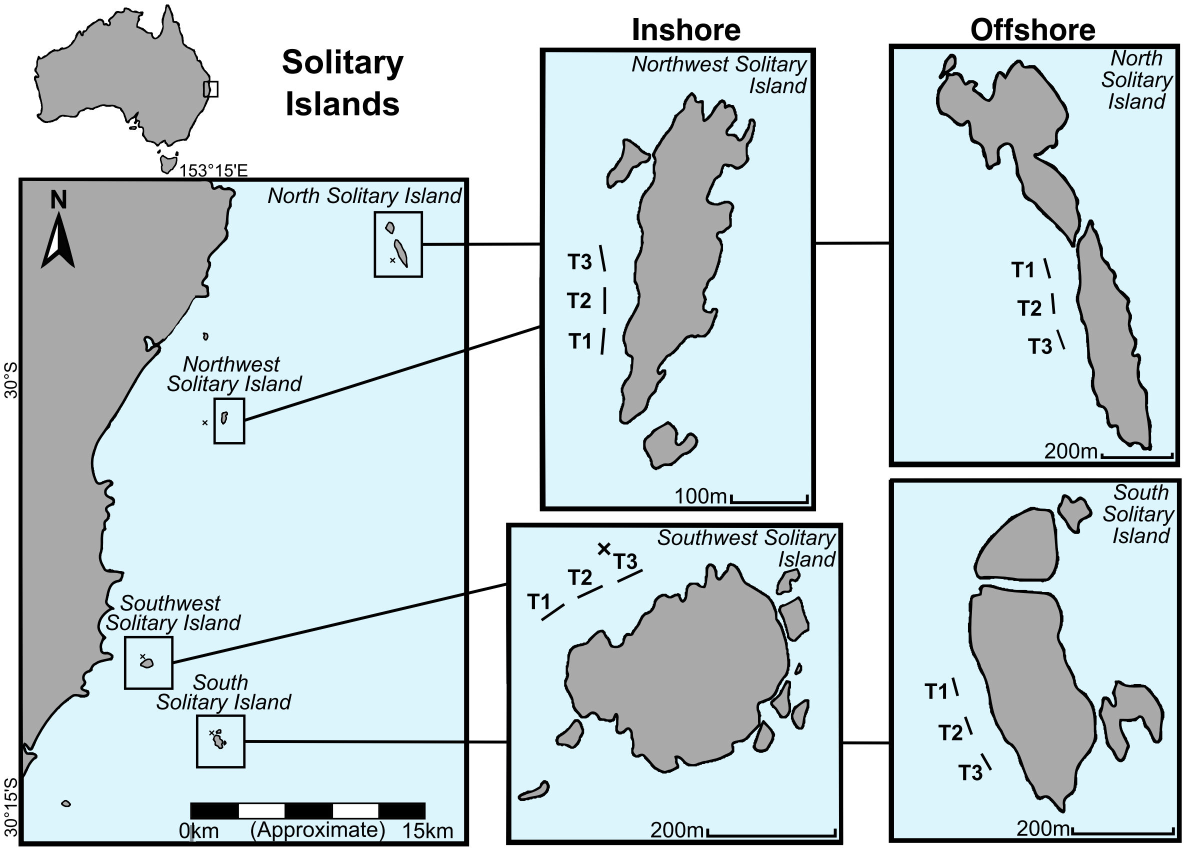

Figure A.1. Map showing the Solitary Islands Marine Park, New South Wales, eastern Australia. Insets mark the sampling sites on the western side of the islands with approximate locations of transects (T1-T3). Offshore islands: North Solitary Island, South Solitary Island; Inshore islands: Northwest Solitary Island, Southwest Solitary Island. Adopted from [1].

Table A.1. Summary of the size-frequency distributions for *Acropora*, *Pocillopora* and *Turbinaria* corals at inshore and offshore sites in the Solitary Islands Marine Park. Mean log colony size, coefficient of variation and skewness calculated from log transformed data.

Taxa	Period	Positon	Number of colonies	Mean colony size (cm^2)	Mean log colony size (cm^2)	Coefficient of Variation	Skewness
Acropora	Period 1	Inshore	446	396.71	4.62	0.38	0.13
		Offshore	392	282.36	4.23	0.39	0.40
	Period 2	Inshore	593	687.37	5.52	0.28	-0.12
		Offshore	570	548.76	5.02	0.33	0.09
	Period 3	Inshore	585	908.94	5.63	0.30	-0.16
		Offshore	410	484.35	4.88	0.33	0.33
Pocillopora	Period 1	Inshore	741	64.65	3.12	0.47	0.08
		Offshore	2095	43.98	2.23	0.76	0.59
	Period 2	Inshore	542	105.25	3.60	0.42	-0.05
		Offshore	2089	66.46	3.46	0.36	-0.09
	Period 3	Inshore	451	131.05	3.95	0.36	0.07
		Offshore	1074	64.62	3.39	0.38	0.01
Turbinaria	Period 1	Inshore	1067	672.62	5.30	0.33	-0.27
		Offshore	35	252.88	4.45	0.37	-0.17
	Period 2	Inshore	964	745.73	5.51	0.30	-0.24
		Offshore	101	318.17	4.40	0.41	0.14
	Period 3	Inshore	949	814.72	5.73	0.28	-0.39
		Offshore	91	234.45	4.40	0.33	0.12

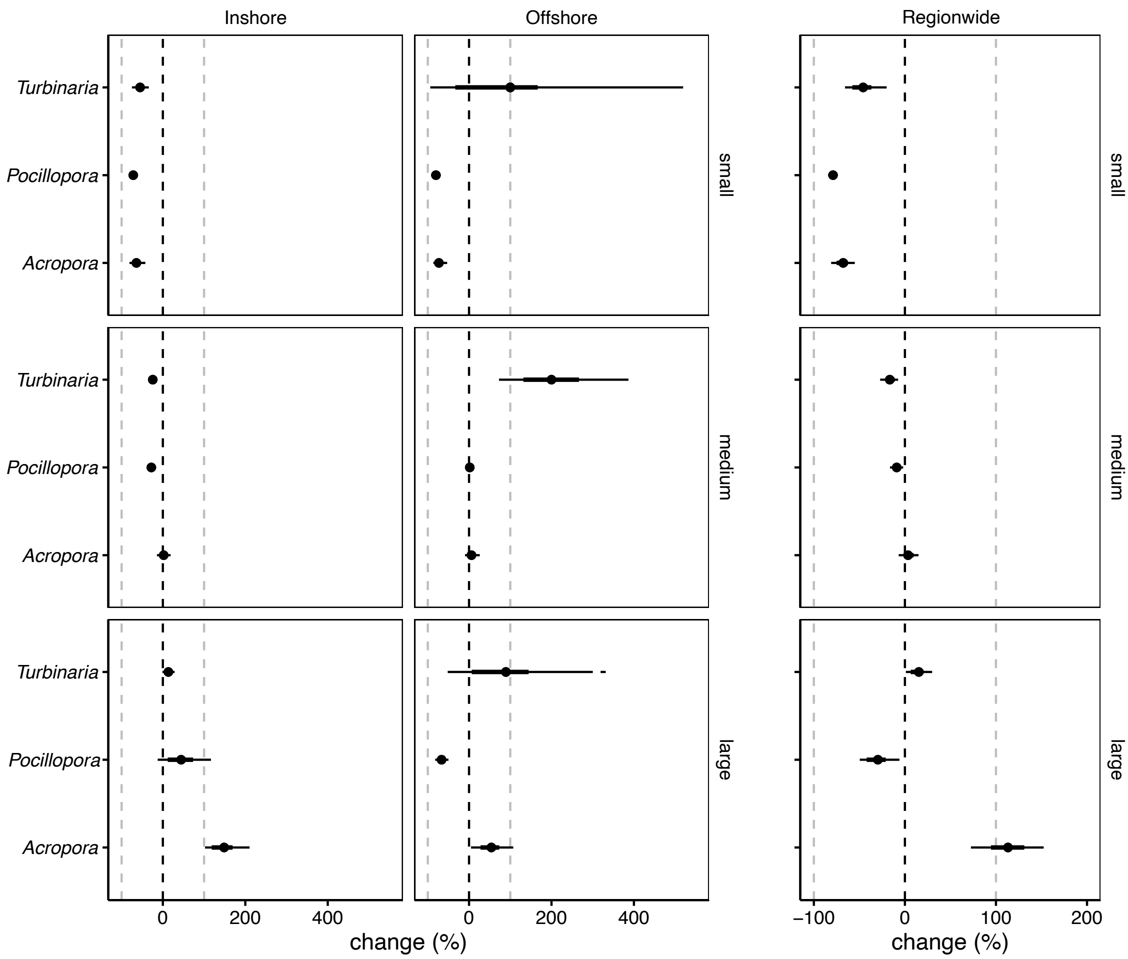

Figure A.2. Changes in the abundance of small, medium, and large colonies for all taxa between for Inshore and Offshore sites, and regionwide between Periods 1 vs 3. Percentage changes in abundances are defined as changes in the number of corals in the 1st quintile (small), 2nd to 4th quintile (medium) and 5th quintile (large) of colony size. All estimates are shown as 95% highest posterior density intervals. The point indicates the median, the thick line the 66% credible interval and the thin line the 95% credible interval.

Table A.2. Spearman rank correlation coefficients for environmental variables. We used a multicollinearity cut-off of r > 0.8 and used DCW_{1C} in our models (and not DCW_{0C}). Coefficients that exceeded r > 0.8 are highlighted in **bold**.

	SST_mean	DCW_{0C}	DCW_{1C}	DHW_{0C}	Chla_mean
SST_mean	1				
DCW_{0C}	0.56	1			
DCW_{1C}	0.78	**0.83**	1		
DHW_{0C}	0.46	**0.81**	0.68	1	
Chla_mean	-0.64	-0.61	-0.58	-0.46	1

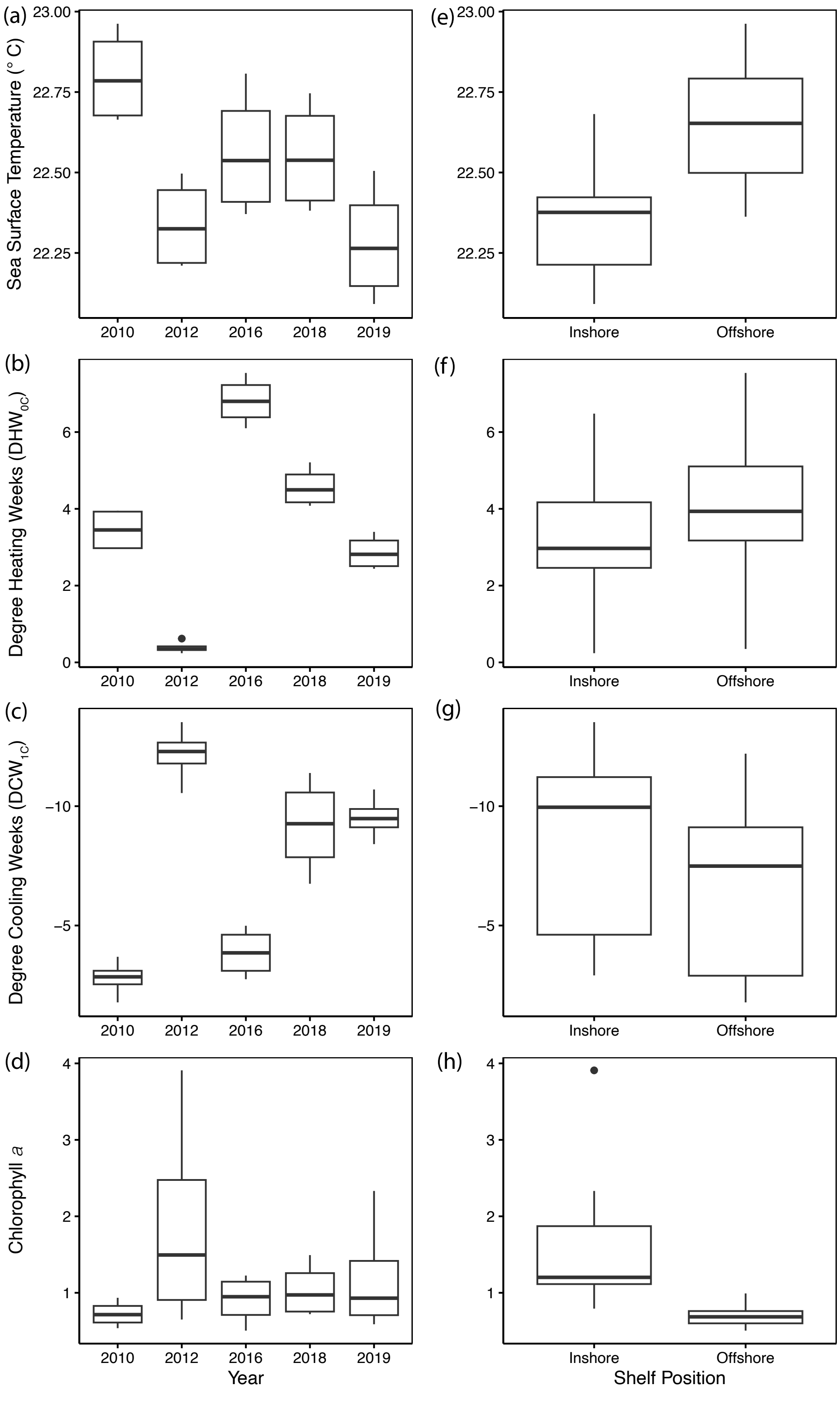

Figure A.3. Environmental conditions in the Solitary Island Marine Park, New South Wales, subtropical Eastern Australia. Boxplots for Mean Sea Surface Temperature (a, e), Degree Heating Weeks (b, f), Degree Cooling Weeks (c, g), and Cholorophyll *a* concentration (d, h) for Years (a-d) and Shelf Position (e-h).

Table A.3. Environmental correlates predicting the abundance of small *Turbinaria*, *Acropora* and *Pocillopora* corals on subtropical reefs, showing R^2, LOOIC, and delta_LOOIC values, as well as loo model weights (weights) from model averaging. Models arrayed from best to worst (top to bottom) for each taxon, with best models in **bold**. SST = mean sea surface temperature; Chla = mean Chlorophyll *a*; DCW_{1C} = Degree Cooling Weeks; DHW_{0C} = Degree Heating Weeks. Blue, red and black shading of environmental predictors represents positive, negative and non-significant relationships, respectively.

Turbinaria	R^2	LOOIC	delta_LOOIC	weights
**Chla**	0.52	112.03		**0.44**
SST + Chla	0.50	113.57	1.5	0.20
SST	0.26	113.62	1.6	0.19
SST + DCW_{1C}	0.33	115.85	3.8	0.06
SST + DHW_{0C}	0.31	116.83	4.8	0.04
DCW_{1C}	0.15	117.10	5.1	0.03
DHW_{0C}	0.13	118.37	6.3	0.02
DCW_{1C} + DHW_{0C}	0.21	120.50	8.5	0.01
Acropora				
**DCW_{1C} + DHW_{0C}**	0.58	102.80		**0.83**
SST + DHW_{0C}	0.51	106.24	3.4	0.16
DHW_{0C}	0.30	114.38	11.6	0.00
SST + Chla	0.34	116.79	14.0	0.00
SST	0.10	118.56	15.8	0.00
DCW_{1C}	0.07	120.20	17.4	0.00
SST + DCW_{1C}	0.16	120.92	18.1	0.00
Chla	0.10	121.04	18.2	0.00
Pocillopora				
**SST + DHW_{0C}**	0.61	194.37		**0.96**
SST + DCW_{1C}	0.38	200.61	6.2	0.04
SST	0.29	211.93	17.6	0.00
DCW_{1C} + DHW_{0C}	0.32	212.31	17.9	0.00
SST + Chla	0.42	216.26	21.9	0.00
DHW_{0C}	0.11	218.06	23.7	0.00
DCW_{1C} + DHW_{0C}	0.07	220.30	25.9	0.00
Chla	0.04	221.02	26.7	0.00

Supplementary results for data that exclude partially captured coral colonies

We recorded a total of 11,990 fully captured coral colonies across 2,160 images; 1,410 partially captured corals were excluded in the following analyses. Sub-setting of the data did not alter the results and conclusions.

Patterns in coral population size structure and abundance

Size-frequency distributions varied among taxa and between Inshore and Offshore habitats (Figs. A.4 and A.5). Mean colony size varied among taxa and was larger inshore for all taxa during all time periods (Fig. A.4). *Pocillopora* corals were more abundant offshore, while *Turbinaria* corals were more abundant inshore (Fig. A.6). Coral population size structure shifted towards larger colonies through time, as shown by increases in mean colony size for all taxa as well as increases in the size of small (20th percentile) and large (80th percentile) colonies and declines in the coefficient of variation (Fig. A.6).

The abundance of small corals declined for all taxa in all periods (Fig. A.7). Although the abundance of medium and large *Pocillopora* and *Acropoca* corals followed a stable to upward trajectory up until 2016 (Periods 1 vs 2), the abundance of medium and large corals declined considerably in the aftermath of coral bleaching (Period 2 vs 3) for all taxa, except for slight increases in the abundance of large *Turbinaria* colonies (Fig. A.7).

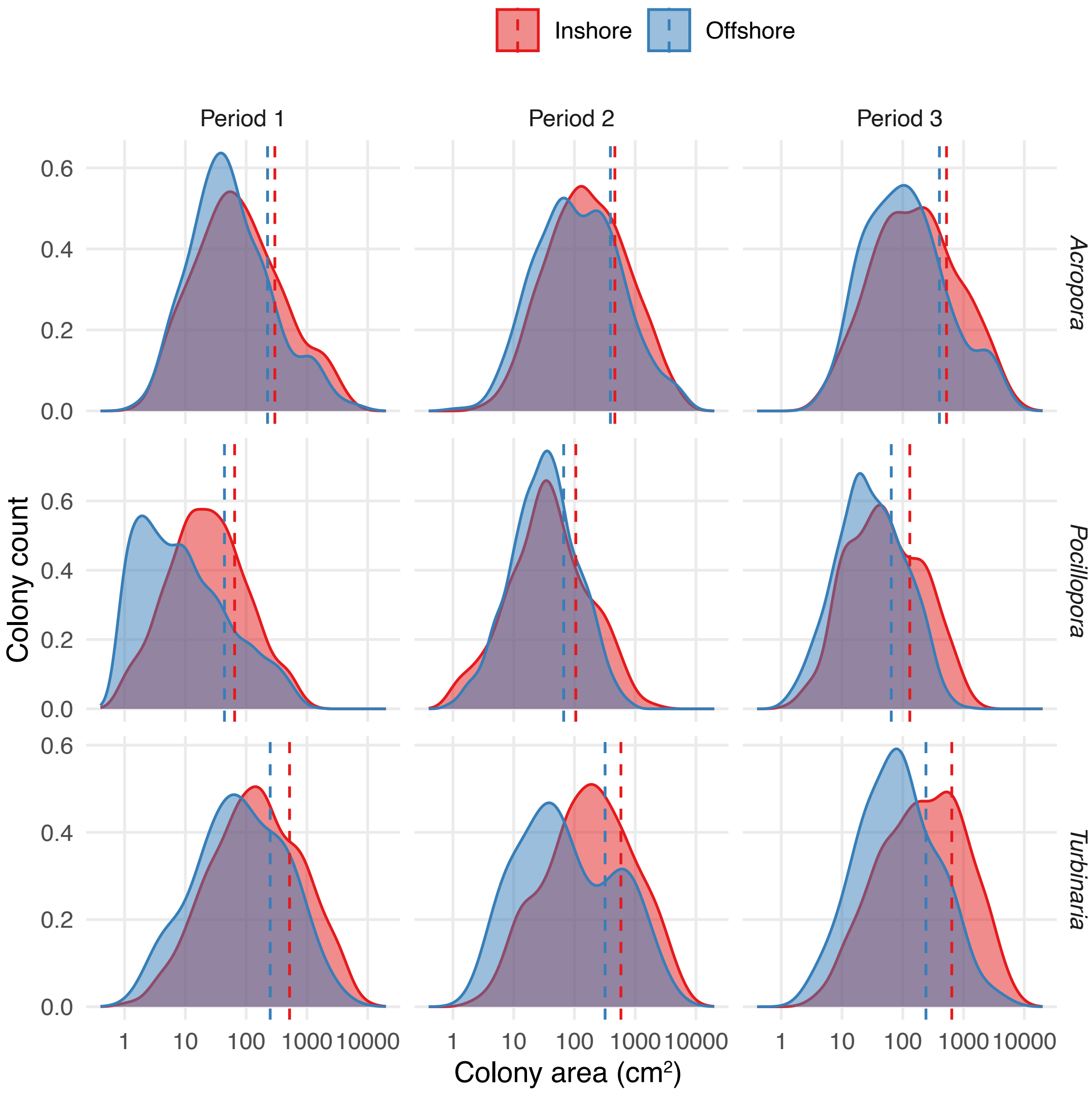

Figure A.4. Size-frequency distributions of colony area for *Acropora*, *Pocillopora* and *Turbinaria* populations at Inshore (red) and Offshore (blue) sites in the Solitary Islands Marine Park in Period 1 (2010, 2012), Period 2 (April and October 2016) and Period 3 (2018, 2019).

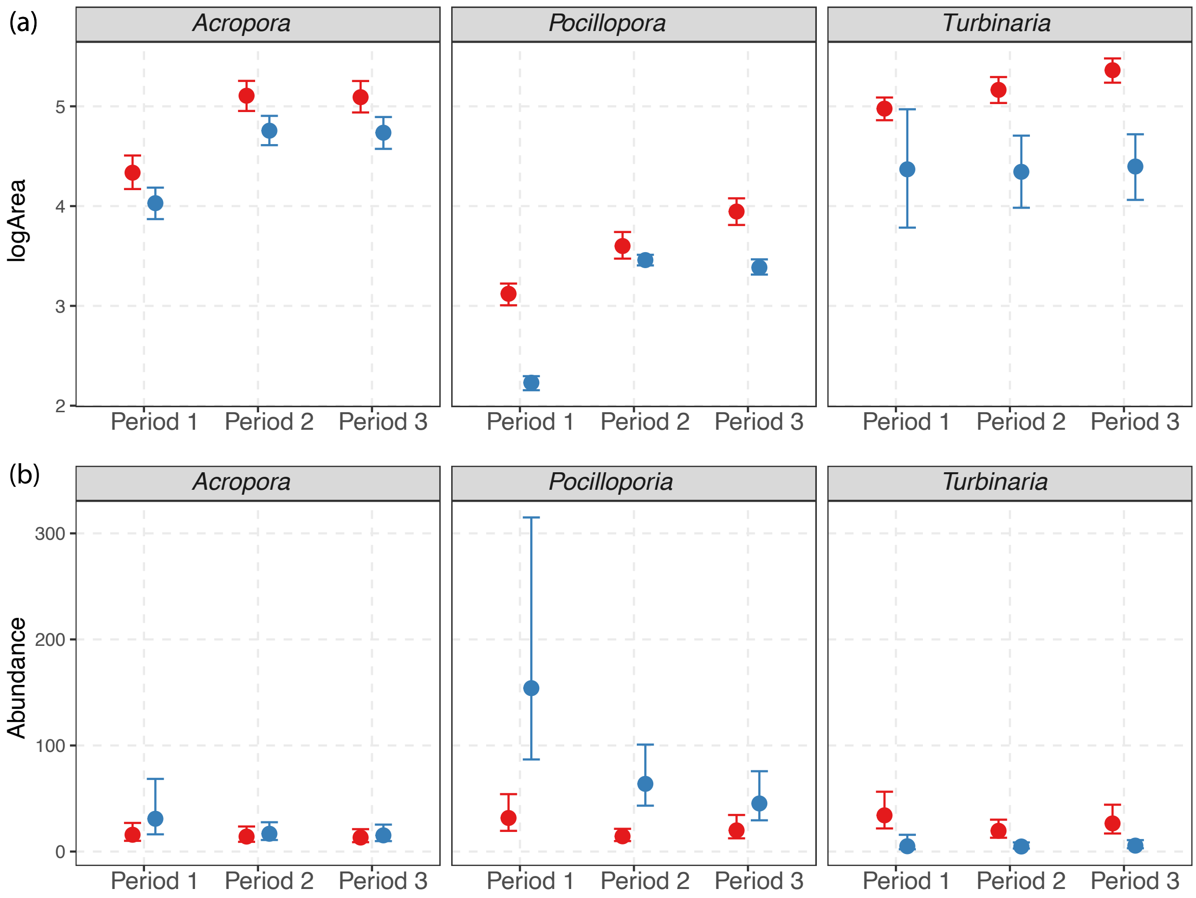

Figure A.5. Patterns in the (a) mean size and (b) abundance of *Acropora*, *Pocillopora* and *Turbinaria* corals at Inshore (red) and Offshore (blue) sites in the Solitary Islands Marine Park in Period 1 (2010, 2012), Period 2 (April and October 2016) and Period 3 (2018, 2019).

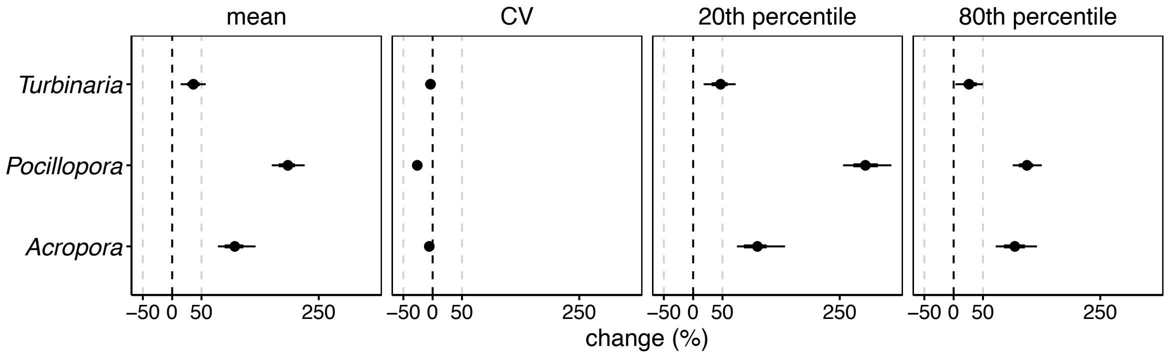

Figure A.6. Changes in the mean, coefficient of variation (CV) and 20th and 80th percentile of the colony size structure of *Acropora*, *Pocillopora* and *Turbinaria* corals in the Solitary Islands Marine Park between Period 1 (2010, 2012) and Period 3 (2018, 2019). Percentiles are indicators for the relative abundance of the smallest (20th percentile) and largest (80th percentile) corals, where increases in the 20th and 80th percentiles indicate a decrease in the relative abundance of the smallest corals and an increase in the relative abundance of largest corals in the population, respectively. All estimates are shown as 95% highest posterior density intervals where the point indicates the median, the thick line the 66% credible interval and the thin line the 95% credible interval.

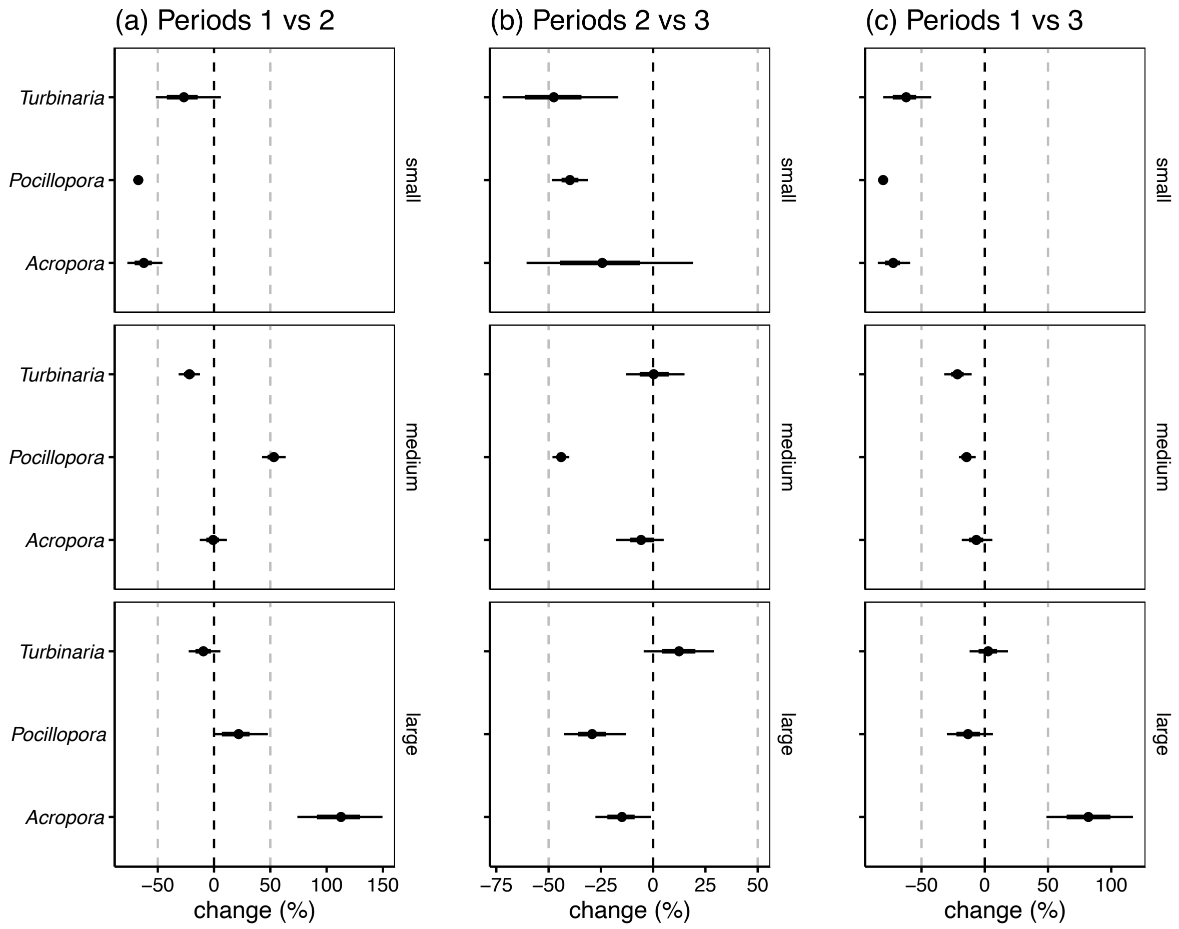

Figure A.7. Changes in the abundance of small, medium, and large colonies for all taxa between Periods 1 vs 2, Periods 2 vs 3, and Periods 1 vs 3. Percentage changes in abundances are defined as changes in the number of corals in the 1st quintile (small), 2nd to 4th quintile (medium) and 5th quintile (large) of colony size. All estimates are shown as 95% highest posterior density intervals. The point indicates the median, the thick line the 66% credible interval and the thin line the 95% credible interval.

Patterns in coral bleaching and partial mortality

The incidence of coral bleaching varied among taxa, with the probability of bleaching highest for *Pocillopora* (0.805), followed by *Turbinaria* (0.622) and minimal bleaching of *Acropora* (0.09). Larger corals had a higher probability of bleaching (Fig. A.8a), and this effect was greatest for *Turbinaria*, followed by *Pocillopora* and was not significant for Acropora (95% CI -0.02, 0.38 overlapped zero).

Coral size also influenced whether a coral suffered partial mortality, with larger *Acropora* and *Pocillopora* corals exposed to greater odds of partial mortality (Fig. A.8b). *Acropora* corals had the highest probability of suffering partial mortality across all periods, followed by *Pocillopora* and *Turbinaria*. The odds of suffering partial mortality were higher in Periods 2 and 3, compared to Period 1 for all taxa (but not significant for *Turbinaria*) (Fig. A.8c).

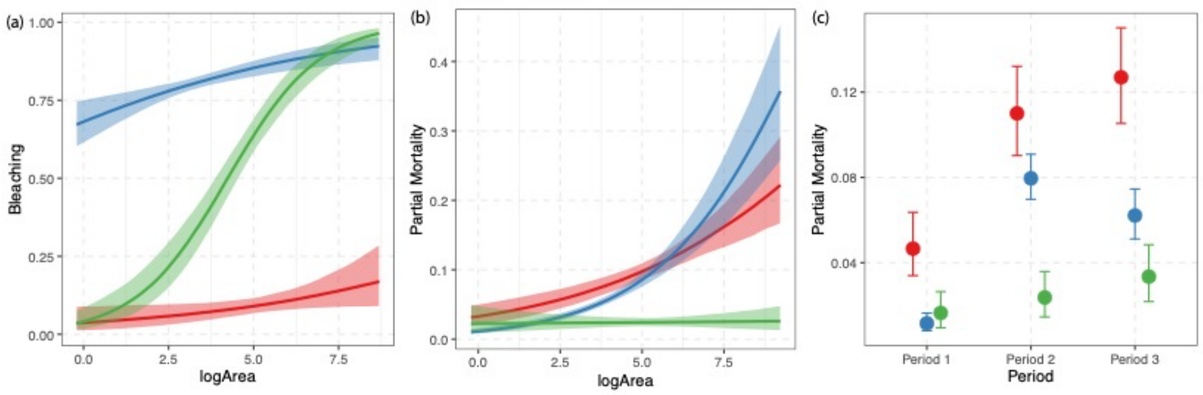

Figure A.8. Relationships between the size of *Acropora* (red), *Pocillopora* (blue), and *Turbinaria* (green) corals and the probability of suffering (a) coral bleaching and (b) partial mortality. (c) Temporal patterns in the probability of partial mortality for Period 1 (2010, 2012), Period 2 (April and October 2016) and Period 3 (2018, 2019).

References

1. Lachs, L. *et al.* Linking population size structure, heat stress and bleaching responses in a subtropical endemic coral. *Coral Reefs* **40**, 777-790, doi:https://doi.org/10.1007/s00338-021-02081-2 (2021).
